# Generation of Highly Purified Human Cardiomyocytes from Peripheral Blood Mononuclear Cell-Derived Induced Pluripotent Stem Cells

**DOI:** 10.1371/journal.pone.0126596

**Published:** 2015-05-13

**Authors:** Maya Fuerstenau-Sharp, Martina E. Zimmermann, Klaus Stark, Nico Jentsch, Melanie Klingenstein, Marzena Drzymalski, Stefan Wagner, Lars S. Maier, Ute Hehr, Andrea Baessler, Marcus Fischer, Christian Hengstenberg

**Affiliations:** 1 Clinic for Internal Medicine II, University Hospital Regensburg, Regensburg, Germany; 2 Department of Genetic Epidemiology, Institute of Epidemiology and Preventive Medicine, University of Regensburg, Regensburg, Germany; 3 Center for and Department of Human Genetics, University of Regensburg, Regensburg, Germany; University of Tampere, FINLAND

## Abstract

Induced pluripotent stem (iPS) cells have an enormous potential for physiological studies. A novel protocol was developed combining the derivation of iPS from peripheral blood with an optimized directed differentiation to cardiomyocytes and a subsequent metabolic selection. The human iPS cells were retrovirally dedifferentiated from activated T cells. The subsequent optimized directed differentiation protocol yielded 30-45% cardiomyocytes at day 16 of differentiation. The derived cardiomyocytes expressed appropriate structural markers like cardiac troponin T, α-actinin and myosin light chain 2 (MLC2V). In a subsequent metabolic selection with lactate, the cardiomyocytes content could be increased to more than 90%. Loss of cardiomyocytes during metabolic selection were less than 50%, whereas alternative surface antibody-based selection procedures resulted in loss of up to 80% of cardiomyocytes. Electrophysiological characterization confirmed the typical cardiac features and the presence of ventricular, atrial and nodal-like action potentials within the derived cardiomyocyte population. Our combined and optimized protocol is highly robust and applicable for scalable cardiac differentiation. It provides a simple and cost-efficient method without expensive equipment for generating large numbers of highly purified, functional cardiomyocytes. It will further enhance the applicability of iPS cell-derived cardiomyocytes for disease modeling, drug discovery, and regenerative medicine.

## Introduction

The groundbreaking discovery that somatic cells can be reprogrammed to a pluripotent state has opened up new avenues for developing more physiologically relevant platforms for drug discovery and toxicity screening, *in vitro* disease models and ultimately even patient-specific cell therapies [1]. While the initial efforts to generate induced pluripotent stem (iPS) cells focused on human fibroblasts as the somatic source for reprogramming, successful generation of iPS cells from other somatic cell types like pancreatic beta cells, gastric epithelial cells, hepatocytes, T and B lymphocytes, keratinocytes, neural progenitor cells and human renal epithelial cells have been reported. [2–9]. Notably, the utilization of blood-derived cells, like T lymphocytes, offers an easy accessible and non-invasive starting material for reprogramming. However, reprogramming efficiencies varies dramatically between different somatic cell types.

Pluripotent stem cells can be turned into cardiomyocytes utilizing either spontaneous or directed differentiation methods. Spontaneous cardiac differentiation can be achieved by using fetal bovine serum containing medium and co-culturing of iPS cells with mouse endoderm-like (END-2) cells [10, 11]. However, these approaches only yield populations of 10% to 25% cardiomyocytes. More recently, directed cardiac differentiation methods mimicking developmental processes during cardiogenesis were developed to direct iPS cells towards a cardiac fate. These protocols are based on media supplemented with certain morphogens and growth factors, such as activin A, bone morphogenic protein 4 (BMP-4), basic fibroblast growth factor (bFGF), vascular endothelial growth factor (VEGF), and dickkopf-related protein 1 (DKK-1) [12–15]. Up to 50% pure cardiomyocytes can be generated employing these differentiation strategies. The remaining so-called contaminating cells consist mainly of fibroblasts, endothelial cells, or smooth muscle cells [16]. In disease model systems, drug testing or regenerative medicine, these mixed or impure cell populations may interfere. Moreover, for regenerative purposes not only large quantities, but also highly purified cardiomyocyte populations are required [17]. Recently, several different strategies for enrichment of cardiomyocytes have been developed. These include the introduction of transgenic selection strategies via drug-selectable elements [18, 19] or fluorescence-activated cell sorting (FACS) with different antibodies [16, 20]. These methods differ largely in their methodological requirements, e.g. genetic manipulation of cells and specialized and expensive instruments. Therefore, only a few laboratories have these methods available. The recently proposed metabolic selection based on media with either reduced glucose or lactate as replacement for glucose provides an easy-to-use alternative [21–23]. These methods exploit the ability of cardiomyocytes to metabolize other energy sources than glucose whereas contaminating cell types dependent on glucose are eliminated.

Here, we report a combined and optimized protocol for the generation of iPS cells from human peripheral blood mononuclear cells with a directed cardiac differentiation approach and subsequent restrictive lactate purification. The validated combination of the above mentioned methods enables the generation of large quantities of highly pure cardiomyocytes as needed for drug testing or regenerative medicine.

## Methods and Materials

### Isolation of PBMCs and T cell expansion

Protocols for derivation of blood samples, reprogramming of human peripheral blood mononuclear cells (PBMCs) to induced pluripotent stem cells (iPS) and subsequent differentiation were in accordance with the Declaration of Helsinki and were approved by the Ethics Committee of Medical Faculty of the University Hospital Regensburg, Germany, under the approval number 11-101-0006. With informed written consent, PBMCs were isolated from whole blood samples derived from a female Caucasian donor by density gradient centrifugation with Ficoll-Paque PREMIUM Reagent (GE-Healthcare) and Leucosep tubes (Greiner bio-one) according to the manufacturer. The isolated PBMCs were either cultivated immediately for T cell proliferation or were cryo-conserved in the gas phase of liquid nitrogen. T cells were expanded as described elsewhere [4]. In brief, PBMCs were cultivated in freshly prepared AIM-V medium (Life Technologies) supplemented with pen/strep and L-glutamine (both from Life Technologies), 300 IU/mL rhIL2 (Peprotech) and 10 ng/mL soluble anti-CD3 antibody (eBioscience) for three days in a 5% C0_2_ atmosphere at 37°C. For reprogramming of frozen PBMCs, the cultivation was prolonged from three to four days until T cell agglomerates were clearly visible. T cell expansion was verified by flow cytometry staining with anti-human CD3 antibody (BD Pharmigen) on a FACS Calibur instrument (BD Pharmigen).

### Retroviral reprogramming of T cells

T cells were reprogrammed with retroviral pMXs-based vectors with human *OCT3/4*, *SOX2*, *KLF4*, *c-MYC* (Addgene plasmids 17217, 17218, 17219, and 17220) as originally developed by Takahashi et al. [1]. Retroviruses were produced by transient transfection of PLAT-A cells (Cell Biolabs, INC.) with single retroviral vectors. In brief, 6 x 10^6^ PLAT-A cells were seeded in T75-cell culture flasks for each vector with DMEM supplemented with 10% FBS, 1 μg/ml puromycin, 10 μg/ml pen/strep (all from Sigma Aldrich) and 10 μg/ml blasticidin S (Life Technologies). The next day, cells were washed with PBS and the transfection-mix was added, containing 10 μg vector DNA, 30 μl Fugene (Promega) in 7 ml DMEM with 10% FBS according to the manufacturer. Retroviral supernatants were collected after 48 h, sterile filtrated with 0.22 μm ultra-low protein binding filter (Millipore) and stored at 4°C until use. 1 x 10^6^ activated T cells were infected by combining 500 μl of each retroviral supernatant in presence of 4 μg/ml polybrene (Millipore) and 300 IU/ml rhIL2 (Peprotech) in 24-well cell culture plates (day 0). After spinfection for 90 min at 1000 rpm, cells were incubated for 4h in a 5% C0_2_ atmosphere at 37°C, followed by a half media exchange with DMEM with 10% FBS and 300 IU rhIL2/ml. The infection procedure was repeated the next day [4]. On day 3, cells were harvested and seeded on MEF-coated 100 mm plates.

### Derivation of iPS cells

**Fig 1:**
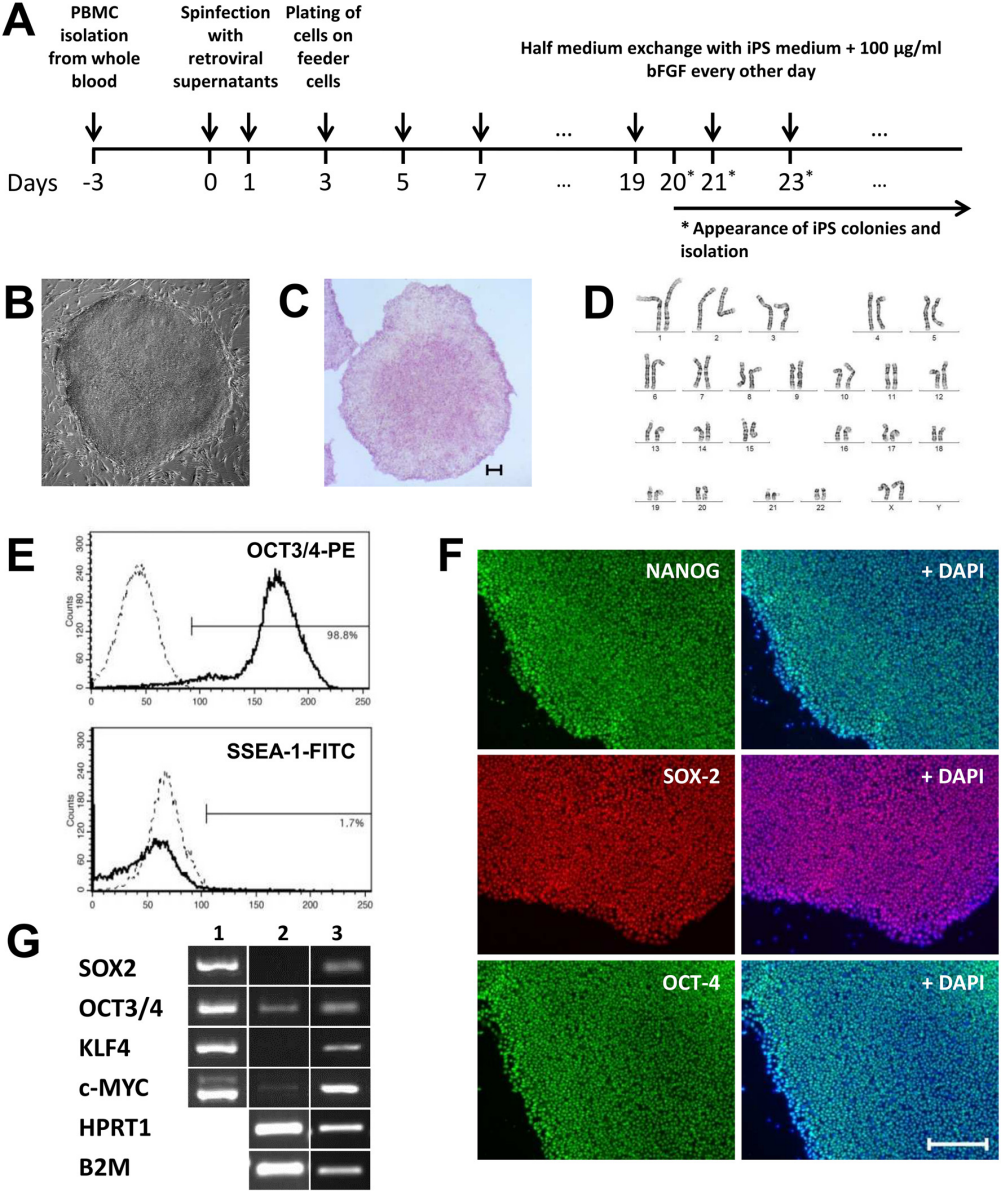
Derivation of iPS cells from PBMCs and their characterization. **A:** Schematic diagram demonstrating the main steps of the reprogramming procedure; **B:** Typical ESC colony appearance; **C:** Positive alkaline phosphatase assay (scale bar 200 μm); **D:** Exclusion of chromosomal aberrations by chromosome analysis (normal female karyotype 46,XX); **E:** Flow cytometry analysis of the marker OCT3/4 (for pluripotency) and SSEA-1 as differentiation marker (dashed lines represent the respective isotype control); **F:** Immunofluorescent staining for the expression of the pluripotency markers NANOG, SOX-2, OCT-4 and overlay with the control staining of the nucleus with DAPI (scale bar 200 μm); **G:** PCR analysis of retroviral integration (1), expression of retroviral (2) and endogenous (3) pluripotency genes with the housekeeping gene controls HPRT1 and B2M.

On day 2 of the reprogramming procedure (Fig 1A) 9 x 10^5^ irradiated CF-1 MEFs (BioChain) were seeded per 100 mm cell culture plate in MEF medium containing DMEM supplemented with 10% FBS and 1x non-essential amino acids (all from Sigma Aldrich). The next day, the plates were washed with PBS and supplied with 7.5 mL fresh MEF medium. Retrovirally infected T cells (day 3) were pelleted, 0.5–2.0 x 10^6^ cells were re-suspended in 7.5 mL iPS medium and added to the prepared MEFs. iPS medium consisted of DMEM/F12, 20% KOSR, 1 mM L-glutamine (all from Life Technologies), 1x non-essential amino acids and 0.1 mM β-mercaptoethanol (Sigma-Aldrich). From day 5 on forward, half medium exchanges were performed every other day with iPS medium supplemented with 100 μg/mL bFGF (Peprotech). Fresh MEFs were added to the plates after day 11 as required. Fully reprogrammed iPS colonies, usually visible between day 20–30, were identified based on their typical hESC morphology (well-defined colony edges, tightly packed cells with prominent nucleoli and a high nucleus to cytoplasm ratio). iPS colonies were isolated and expanded in 12-well or 6-well plates on fresh MEF feeder layers in iPS medium with bFGF. iPS cells were adapted to feeder-free conditions with mTeSR1 (Stemcell Technologies) on Matrigel (BD Biosciences) between passages 3 to 5. Two rounds of retroviral reprogramming were needed to generate 13 high quality iPS cell lines from one donor.

### Characterization of iPS cells

#### Flow Cytometry Analysis

iPS cells were dissociated with accutase (Life Technologies), washed and re-suspended in PBS + 2% BSA for surface staining overnight with the surface antibodies mouse anti-SSEA-1-FITC, rat anti-SSEA-3-PE, and mouse anti-TRA-1-81-Alexa Fluor 647 (Human Pluripotent Stem Cell Sorting and Analysis Kit, BD Bioscience, 10 μL + 100 μL buffer). For the intracellular marker mouse anti-Oct3/4-PE (BD Biosciences, 10 μL + 90 μL buffer) iPS cells were treated with the Cytofix/Cytoperm Kit (BD Biosciences) according to the manufacturer. The next day, cells were washed and analyzed on a flow cytometer.

#### Immunocytochemical analysis

For intracellular staining, iPS cell colonies were fixed and permeabilized using the Cytofix/Cytoperm Kit (BD Biosciences) according to the manufacturer’s recommendation. iPS colonies were overnight stained with directly conjugated mouse anti-OCT4 Alexa Fluor 488 (Millipore, 1:100) mouse anti-NANOG Alexa Fluor 488 (Millipore, 1:100) and unconjugated mouse anti-SOX-2 (Millipore 1:100). The next day, secondary antibody staining with Alexa Fluor 647 (Life Technologies, 1:500) was performed on iPS colonies previously stained with unconjugated SOX-2 antibody. Subsequently, all iPS colonies were also stained with DAPI (NucBlue Fixed Cell Stain, Life Technologies) prior to fluorescent microscopic analysis. Besides assessing the presence of typical intracellular pluripotency markers, iPS colony surface marker detection was carried out using primary unconjugated mouse anti-TRA-1-60 (Millipore, 1:100) and mouse anti-TRA-1-81 (Millipore, 1:200) followed by secondary antibody staining with Alexa Fluor 488 (Life Technologies, 1:500) and DAPI live cell staining (NucBlue Live Cell Stain, Life Technologies) (data not shown). Fluorescence was monitored with a Zeiss Observer Z1 microscope.

#### Alkaline phosphatase assay

The alkaline phosphatase (AP) assay was performed according to the protocol provided by the manufacturer (Stemgent). In brief, iPS cells grown in 6-well plates were washed with PBS prior to fixation at room temperature. After fixation, the cells were washed again before applying freshly prepared AP substrate solution followed by incubation at room temperature protected from light. The staining reaction was stopped by aspirating the AP substrate solution and washing the cells with PBS. Monitoring of the staining reaction was performed with a Zeiss Observer Z1 microscope.

#### Karyotyping

iPS cells were plated on Matrigel-coated T25 flasks and cultivated for 2–3 days in mTeSR1 medium. Chromosome analysis was carried out according to established protocols. In brief, cells were synchronized using thymidine solution (Sigma-Aldrich) and subsequently treated with colcemid (Roche) for 10 min at 37°C. After detachment with trypsin—EDTA, cells were centrifuged, and the cell pellet was re-suspended and maintained in hypotonic solution (75 mM KCl) for 12 min at 37°C. Cells were then fixed in methanol and acetic acid. Metaphase spreads were prepared on cover slips, dried overnight and Giemsa stained (Sigma-Aldrich) after trypsin pre-treatment.

#### PCR and RT-PCR analysis

Genomic DNA was isolated from iPS cells using the AllPrep DNA/RNA Mini Kit (Qiagen) according to the manufacturer’s protocol. The primers used to confirm retroviral vector integration were originally designed by Takahashi et al. [1]. 50 ng of genomic DNA was used as templates for PCR with HotStarTaq Master Mix Kit (Qiagen) at a final volume of 25 μl. PCR products were electrophoretically separated in a 2% agarose gel. Total RNA from iPS cells was isolated using the RNeasy Mini Kit (Qiagen) according to the manufacturer. Subsequently, cDNA was made using the Affinity Script cDNA Synthesis Kit (Agilent). To detect the expression of the endogenous pluripotency genes *OCT3/4*, *SOX2*, *KLF4* and *c-MYC*, primers originally designed by Takahashi et al. [1] were used. Primer assays from Qiagen were used for the housekeeping genes B2M and HPRT1. RT-PCR was performed with 10 ng RNA-equivalent and HotStarTaq Master Mix as described above.

### Cardiac Differentiation of iPS cells

iPS cells were dissociated with accutase followed by centrifugation at 1.200 rpm for 5 minutes. Cell pellets were then re-suspended in mTeSR supplemented with 1 μM H1152 (Tocris) and 50 mg/mL of gentamicin (Sigma Aldrich) at a cell density of 1 x10^6^ cells/mL [4]. For subsequent differentiation, the cell suspension was transferred to ultra-low attachment flasks (BD Bioscience) and cultured in a 5% C0_2_ atmosphere at 37°C. T25 ultra-low attachment flasks were filled with 5 ml cell suspension, T75 flasks with 15 ml. On day 1 of differentiation, half of the medium was removed and replaced with fresh medium containing 50% mTeSR, 45% DMEM (low glucose), 5% FBS, 1 μM H1152, 100 ng/mL of bFGF and 25 μg /mL of gentamicin. The flasks were then returned to the incubator, placed on an orbital shaker. The next day, 2/3 of the medium was replenished with DMEM (low glucose) supplemented with 10% FBS, 50 ng/mL of bFGF and 25 μg/mL of gentamicin. From day 3 to day 7 of differentiation, medium replenishments occurred daily using DMEM (low glucose) with 10% FBS, 50 ng/mL of bFGF and 25 μg/mL of gentamicin. In addition, during this time period, the differentiation medium contained 6 ng/mL of activin A and 10 ng/mL of BMP-4 [12]. From day 8 of differentiation, the cardiac clusters were fed every other day with DMEM (low glucose) supplemented with 10% FBS and 25 μg/mL of gentamicin. Starting on day 14 of differentiation, the cardiac aggregates were cultured at 20% O_2_ and 7% CO_2_. Cardiac clusters usually began to spontaneously beat between day 9 and 13 of differentiation. Flow cytometry analysis to detect the expression of cardiac-specific markers was performed between day 16 and 18 of differentiation.

### Purification of iPS cell-derived cardiomyocytes

#### Purification using magnetic-activated cell sorting (MACS)-based positive selection

For magnetic-activated cell sorting (MACS)-based positive selection selection, PE conjugated anti-CD172a (SIRPA, BioLegend) and anti-CD106 (VCAM1, BioLegend) antibodies were used [16, 20]. Cardiac aggregates were dissociated with 10x trypsin (Sigma Aldrich), run through a 30 μm pre-separation filter and centrifuged. The resulting pellet was re-suspended in 300 μL of MACS buffer (Miltenyi Biotec). For positive selection, 1 x 10^6^ cells were labeled with 5 μl of CD172a or 20 μl of CD106 PE conjugated antibody for 20 min followed by incubation with anti-PE-beads (Miltenyi Biotec) for additional 15 min. Next, the cells were washed by adding 2 mL of MACS buffer per 1x10^7^ cells followed by centrifugation. The pellet was then re-suspended in 80 μL of MACS buffer per 1x 10^7^ cells and 20 μL of anti-PE-micro beads (Miltenyi Biotec). Incubation for 15 minutes at 4°C followed. In an additional wash step, 2 mL of MACS buffer was added per 1x 10^7^ followed by centrifugation. The resulting pellet was re-suspended in 500 μL of MACS buffer and dropped onto a primed MACS MS column (Miltenyi Biotec). After washing the column three times with MACS buffer, the MACS purified cells were recovered from the column using a plunger. CD172a or CD106 positive cells were pelleted, re-suspended in DS-CMM, counted, plated and analyzed by flow cytometry.

#### MACS-based depletion

To deplete contaminating cells, such as fibroblast, smooth muscle cells and endothelial cells from the cultures, CD90 microbeads (Miltenyi Biotec) and PE labeled anti-CD140b (BD Pharmingen) were used as negative surface markers [16]. Magnetic labeling with anti-PE micro beads was performed as described for the MACS-based positive selection. To isolate the labeled cardiomyocytes the cells were run through an LD column (Miltenyi Biotec). After several washes with MACS buffer, the flow-through containing CD90 and CD140b negative cells was collected, pelleted, re-suspended in DMEM supplemented with 10% FBS counted, plated and analyzed by flow cytometry.

#### Metabolic selection with lactate

From day 16 to 18, the cultures were fed with lactate medium composed of DMEM (no glucose) supplemented with 1% sodium DL-lactate solution (60%, Sigma Aldrich) and 25 μg/mL of gentamicin [22]. For the first 2 days of purification, the medium was replenished daily. Thereafter, medium exchanges occurred every other day. Lactate medium-based purification was performed for a maximum of 7 days. Cardiac clusters were then dissociated with 0.5% trypsin (Sigma Aldrich) and processed for flow cytometry analysis to determine the amount of cardiac troponin T (cTnT) positive cells as a marker for cardiomyocyte content. Subsequently, the cells were plated in DMEM with 10% FBS and 25 μg/mL of gentamicin onto 0.1% gelatin-coated plates. After 2 days in culture, cultures containing ≥ 90% cTnT positive cells on the day of dissociation were maintained in DMEM (low glucose) supplemented with 2.5% FBS and 25 μg/mL of gentamicin until further analysis. Cultures yielding ≤ 90% cTnT positive cells after 7 days of treatment with lactate medium were subjected to additional lactate medium-based purification for 3–5 days before they were also switched to DMEM (low glucose) supplemented with 2.5% FBS and 25 μg/ml gentamicin.

### Cardiomyocyte characterization

#### Flow cytometry

Cardiac aggregates were dissociated with 0.5% trypsin followed by fixation and permeabilization using Cytofix/Cytoperm reagent (BD Biosciences) according to the manufacturer. Cell were then stained with primary mouse anti-cTnT (Abcam, 1:500) overnight followed by cardiomyocytes staining with the secondary antibody, goat anti-mouse IgG1 Alexa488 (Life Technologies, 1:500). After 1h of secondary antibody incubation, the cells were washed and assayed on a flow cytometer.

#### Immunocytochemical analysis

In preparation for immunofluorescent staining, plated cardiomyocytes were washed once with PBS. The cells were fixed and permeabilized by applying Cytofix/Cytoperm für 20 minutes. After fixation, the cells were washed with Permwash (BD Biosciences) and stained with primary antibody dilutions for rabbit anti-cardiac troponin T (Abcam, 1:200) mouse anti-α-actinin (Abcam, 1:1000), rabbit anti-myosin light chain 2 (MLC2v) (Proteintech, 1:200), rabbit anti-connexin 43 (Abcam, 1:1000) and rabbit anti-N cadherin (Abcam, 1:200) overnight. The next day, the cells were washed and stained with the respective secondary antibodies anti-rabbit IgG Alexa594, goat anti-mouse IgG1 Alexa488, donkey anti-rabbit IgG Alexa488 or goat anti-mouse IgG1 Alexa594 (all Life Technologies, 1:1000). Next, the stained cardiomyocytes were washed, stained with DAPI to visualize nuclei and evaluated with a Zeiss Observer Z1 microscope.

#### Quantitative PCR analysis to assess cardiac specific marker expression

RNA from iPS cell derived cardiomyocytes was isolated using the Ambion Cells-to-C_t_ Kit (Life Technologies) according to the manufacturer. Cardiomyocytes were dissociated as described above. To quantify the expression of pan-cardiac marker cardiac troponin T (cTnT) and ventricular specific marker myosin light chain 2 (MYL2) during cardiac differentiation, quantitative polymerase chain reaction (QPCR) was performed using pre-designed TaqMan assays (S1 Table). QPCR reagents were assembled in 384-well plates using 10 μL Taqman Gene Expression Mastermix, 5 μL of nuclease free water, 1 μL of the Taqman Gene Expression Assay of choice and 4 μL of cDNA. The PCRs were run on a ViiA7 real time instrument (Life Technologies) using the following program: 2 minutes at 50°C, 10 minutes at 95°C, 40 cycles of 95°C for 15 seconds and 1 minute at 60°C. Relative gene expression was calculated with the ΔΔCt method normalizing target gene expression to B2M [24].

#### Patch-clamp experiments

Ruptured-patch whole-cell voltage-clamp was used to measure membrane potential in current clamp configuration as previously described [25–29]. Briefly, cardiomyocytes derived from iPS cells were mounted on the stage of a microscope (Zeiss Axiovert). Microelectrodes (4 MΩ) were filled with 120 mmol/L K-aspartate, 8 mmol/L KCl, 7 mmol/L NaCl, 1 mmol/L MgCl_2_, 10 mmol/L HEPES, 5 mmol/L Mg-ATP, 0.3 mmol/L Li-GTP, 1 mmol/L EGTA, 0.2 mmol/L CaCl_2_ (free [Ca^2+^]_i_ 40 nmol/L) (pH 7.2, KOH). The bath solution contained 140 mmol/L NaCl, 4 mmol/L KCl, 1 mmol/L MgCl_2_, 2 mmol/L CaCl_2_, 10 mmol/L Glucose, 5 mmol/L HEPES (pH 7.4, NaOH). Access resistance was typically ~20 MΩ after patch rupture. Spontaneous action potentials (APs) were recorded immediately after patch rupture and followed for about 2 min. Signals were filtered with 2.9 kHz and 10 kHz Bessel filters, and recorded with an EPC10 amplifier (HEKA Elektronik, Lambrecht/Pfalz, Germany) using the Patchmaster software. At least 10 action potentials in a row were averaged and analysis was done using LabChart Pro software (ADInstruments) to determine the maximum rate of rise of the AP upstroke (V_max_), AP amplitude (APA), the AP durations (APDs) at 30, 50, 80, and 90% of repolarization (APD30, APD50, APD80, APD90) and the resting membrane potential (RMP). For some experiments, APs were continuously elicited by square current pulses of 1–2 nA amplitude and 1–5 ms duration at various basic cycle lengths. All experiments were conducted at room temperature.

#### Intracellular calcium measurements using confocal laser microscopy

Intracellular calcium ([Ca^2+^]_i_) signals were recorded after incubating cells with 10 μmol/L fluo-4 acetoxymethylester (Life Technologies) for 30 min on a laser scanning confocal microscope (Zeiss LSM 700) as previously described [30]. Cells were washed with Tyrode’s solution. Fluo-4 was excited via an argon laser (488 nm, 10 mW) and emitted fluorescence (F) was separated by a variable secondary dichroic beam splitter (at 492 nm) and a 500 nm long-pass emission filter. Changes in fluo-4 fluorescence (indicating fluctuation in cytosolic Ca^2+^) were recorded in frame and line scan mode while the cells were beating spontaneously. The images were acquired and analyzed using Zeiss software and Image J. Fluorescence signals were normalized to basal cell fluorescence after fluo-4 loading (F_0_) and converted into [Ca^2+^]_i_ by the following pseudo-ratio equation [31]: [Ca]_i_ = K_d_(F/F_0_)/(K_d_/[Ca]_i-rest_ + 1–F/F_0_) with K_d_ = 1100 nmol/L and [Ca]_i-rest_ = 100 nmol/L. In some experiments isoproterenol (1 nmol/L) was added to the bath solution during recording. All experiments were conducted at room temperature.

#### Fluorescence recovery after photobleaching (FRAP) assay

Functional analysis of gap junctions in isolated cardiac clusters was performed by measuring the cell-to-cell diffusion of a fluorescent dye using a FRAP assay at room temperature. Briefly, cardiac clusters were loaded with the membrane-permeant fluorescent dye fluo4-AM (5 μmol/L; Invitrogen) in Ca-free Tyrode solution for 20 min at 37°C. After washing away the excess extracellular fluorescent dye to prevent further loading, the cultures were bathed in Ca-free Tyrode solution and placed on the stage of a Zeiss LSM 700 laser-scanning confocal microscope. Using Zeiss software, a rectangular region encompassing 95% of a single cell within a cell cluster was selected and its fluorescence was bleached by a high-intensity laser pulse (488 nm, 3–5 s duration). This caused immediate loss of fluo-4 fluorescence emission recorded through a 500 nm long-pass emission filter after separation by a variable secondary dichroic beam splitter (at 492 nm). Fluo-4 redistribution from adjacent unbleached cells through connexin pores into the bleached region of interest (ROI) was recorded in subsequent confocal images acquired at 30 s intervals for up to 10 min. Fluorescence recovery within the ROI was plotted as a function of time and fit to a single exponential function: I_ROI_ (t) = A [1 - exp^-kt^], where I_ROI_ (t) is the ROI fluorescence intensity at time t, A is the amplitude of fluorescence recovery, and k is the rate of recovery. The latter is considered a measure of gap junction permeability [32].

## Results

PBMCs were isolated directly after the blood samples were taken and were retrovirally reprogrammed. iPS cell colonies started to appear 20 days post initial transduction of T cells with the retroviral transcription factors and were isolated between 23 and 27 days after reprogramming (Fig 1A). All iPS colonies were initially identified based on morphological characteristics, such as well-defined colony edges, tightly packed cells with prominent nucleoli and a high nucleus to cytoplasm ratio (Fig 1B). Based on this assessment, a total of 13 iPS colonies were isolated from the donor followed by expansion, characterization and cryo preservation (banking).

### iPS cell characterization

To determine the quality of the generated iPS cell lines, alkaline phosphatase (AP) activity was assessed. This test showed that AP is expressed uniformly high throughout the iPS colonies (Fig 1C). Additionally, chromosome analysis was performed confirming a normal karyotype for all iPS cell lines tested (Fig 1D). Flow cytometry analysis for the pluripotency marker Oct-3/4 as well as the differentiation marker SSEA-1 revealed that all generated iPS cell lines were > 95% positive for Oct-3/4 and ≤ 5% positive for SSEA-1 indicative of high quality iPS cells (Fig 1E). In addition, immunocytochemistry staining for the pluripotency markers NANOG, SOX-2 and OCT-4 demonstrated that the iPS colonies uniformly expressed high levels of these markers (Fig 1F). Lastly, integration of the retroviral reprogramming vectors and their silencing as well as high levels of endogenous *OCT-4*, *KLF-4*, *SOX-2* and *c-MYC* gene expression could be confirmed (Fig 1G).

### Cardiac differentiation

**Fig 2:**
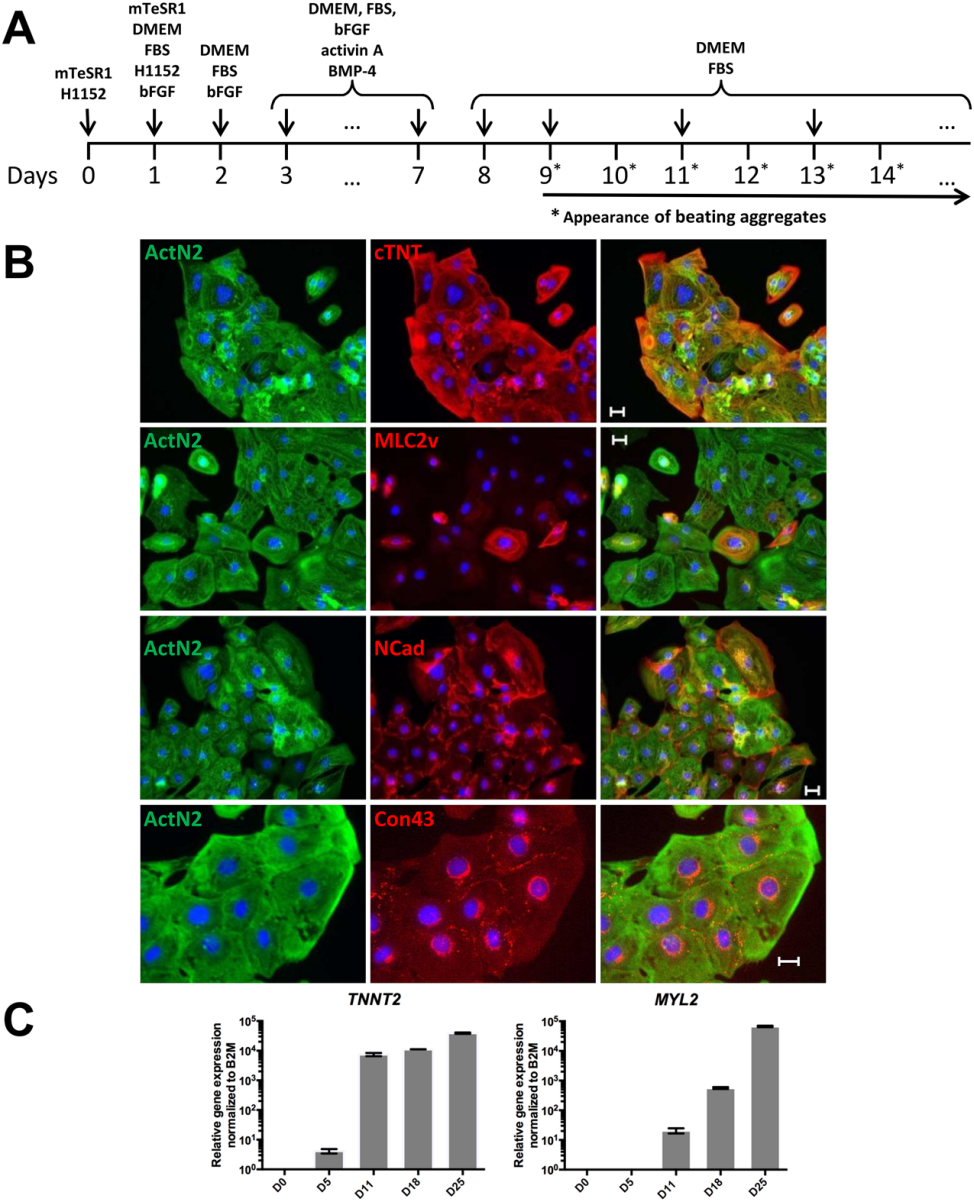
Differentiation and characterization of iPS cell-derived cardiomyocytes. **A:** Schematic diagram demonstrating the main steps of the procedure used for the direct differentiation of iPS cells to cardiomyocytes. The main components of the media applied are noted above the time line. Cell suspensions were kept on an orbital shaker at 5% O2 and 7% CO2 from day 1 to 14. Then the flasks with the cell aggregates were moved to an orbital shaker at 20% O2 and 7% CO2; **B:** Immunofluorescent staining of cardiomyocytes with α-actinin (ActN2), cardiac troponin T (cTNT), myosin light chain 2 (MLC2v), and N cadherin (NCad)(scale bar 25 μm), cardiac troponin T and connexin 43 (Con43)(scale bar 50 μm); **C:** TNNT2 and MYL2 gene expression in iPS cell-derived cardiomyocytes. Gene expression for both genes increases until day 25 of differentiation.

Quality controlled iPS cells were then subjected to cardiac differentiation as depicted in Fig 2A. Three different seeding densities (500,000 cells/mL, 1 x 10^6^ cells/mL and 1.5 x 10^6^ cells/mL) for aggregate formation on day 0 of differentiation were initially tested across several iPS cells lines by evaluating aggregate morphology and quantity. This test revealed that a starting seeding density of 1 x 10^6^ cells/mL was optimal for most cell lines as it resulted in homogeneous aggregate populations.

The differentiation cocktail comprised 50 ng/mL of bFGF, 6 ng/mL of activin A and 10 ng/mL of BMP-4. Initially, cardiac differentiation was carried out at 20% CO_2_ resulting in variable differentiation efficiencies (data not shown). However, once hypoxic conditions were implemented by lowering O_2_ to 5%, the efficiencies became more robust. In brief, iPS cells were differentiated into cardiomyocytes at 5% O_2_, 7% CO_2_ for the first 14 days of differentiation followed by maintenance at 20% O_2_, 7% CO_2_ thereafter. Overall, using a starting seeding density of 1 x 10^6^ iPS cells/mL in combination with 50 ng/mL of bFGF, 6 ng/mL of activin A and 10 ng/mL BMP-4 from day 3 until day 7 of differentiation followed by factor withdrawal from day 8 on forward under hypoxic growth conditions from day 0 to day 14 of differentiation reproducibly resulted in 30–45% pure cardiomyocytes by day 16 of differentiation. This result was obtained across different iPS cell lines as determined by flow cytometry analysis for cTnT expression (Table 1, S2 and S3 Tables). Beating aggregates routinely appeared from day 9 to 12 of differentiation (Fig 2A, S1 and S2 Movies).

**Table 1:** Cardiomyocyte yields after enrichment by MACS or purification with lactate metabolic selection.

	before enrichment/purification			after enrichment/purification			cells lost in %	
	total cells	% cTNT+	cTNT+ cells	total cells	% cTNT+	cTNT+ cells	total cells	cTNT+ cells
MACS positive selection (SIRPA)	9.4 x 10^6^	36.0	3.4 x 10^6^	9.0 x 10^5^	80.2	7.2 x 10^5^	90.4	78.6
MACS positive selection (VCAM1)	6.8 x 10^6^	36.3	2.5 x 10^6^	5.0 x 10^5^	89.1	4.5 x 10^5^	92.6	82.0
MACS depletion (CD90+CD140b)	9.3 x 10^6^	23.3	2.2 x 10^6^	1.4 x 10^6^	49.8	6.8 x 10^5^	85.2	68.3

Cell counts and percentages shown are from single representative direct differentiations. Detailed information including data from several enrichments or purifications can be found in S2 and S3 Tables. Total cell counts and cTnT positive cell counts (cTnT+ cells) were calculated per T75 flask.

### Investigation of cardiac-specific expression markers

Immunocytochemistry of plated cardiomyocytes was subsequently performed to assess the expression of the cardiac-specific markers cTnT, sarcomeric α-actinin and myosin light chain 2 (MLC2v) as well as connexin 43 and N cadherin. Co-expression of sarcomeric α -actinin with cTnT, MLC2v as well as N cadherin and connexin 43 could be detected indicative of the presence of cardiomyocyte-specific sarcomeric and cytoskeletal structures (Fig 2B).

To characterize the iPS cell-derived cardiomyocytes for the expression of the cardiac specific genes *TNNT2* and *MYL2*, TaqMan-based gene expression studies were performed. They showed that the expression of the pan-cardiac marker *TNNT2* increased as beating aggregates appeared in the cultures (Fig 2C, left panel). *TNNT2* expression continued to rise until day 25 of culturing. *MYL2* expression was first detected on day 11 of differentiation and increased thereafter (Fig 2C, right panel). During cardiac development as well as in the adult human heart, the expression of this marker is restricted to the ventricles [33].

### Application of different purification strategies

Initially, MACS-based purification was attempted since several recent publications reported the successful enrichment of cardiomyocytes based on this strategy. Purification of cardiac populations utilizes either SIRPA (CD172a) or VCAM1 (CD106) surface marker expression while depletion of contaminating cell types relies on THY1 (CD140b) and PDGFRB (CD90) expression. These markers are expressed on smooth muscle cells and fibroblasts respectively. Using either positive or negative selection, enrichment of cardiomyocytes by 2-fold could be achieved as determined by subsequent flow cytometry analysis for cTnT (Table 1 and S2 Table). However, the final purities were still below 90%. In addition, the flow through contained 20–30% cTnT positive cells (data not shown), indicating that the MACS column did not sufficiently bind all labeled cardiomyocytes. Another drawback of MACS based purification by positive selection with cardiomyocyte-specific antibodies or depletion of contaminating cells was that 80–90% of total cells and 70–85% of cardiomyocytes were routinely lost in the process (Table 1 and S2 Table), e.g. to obtain 1x 10^6^ cardiomyocytes, an initial input of 13–15 x 10^6^ cells was needed. Finally, after plating on gelatin-coated plates, the purified cardiomyocytes did not attach well resulting in additional cellular losses.

**Fig 3:**
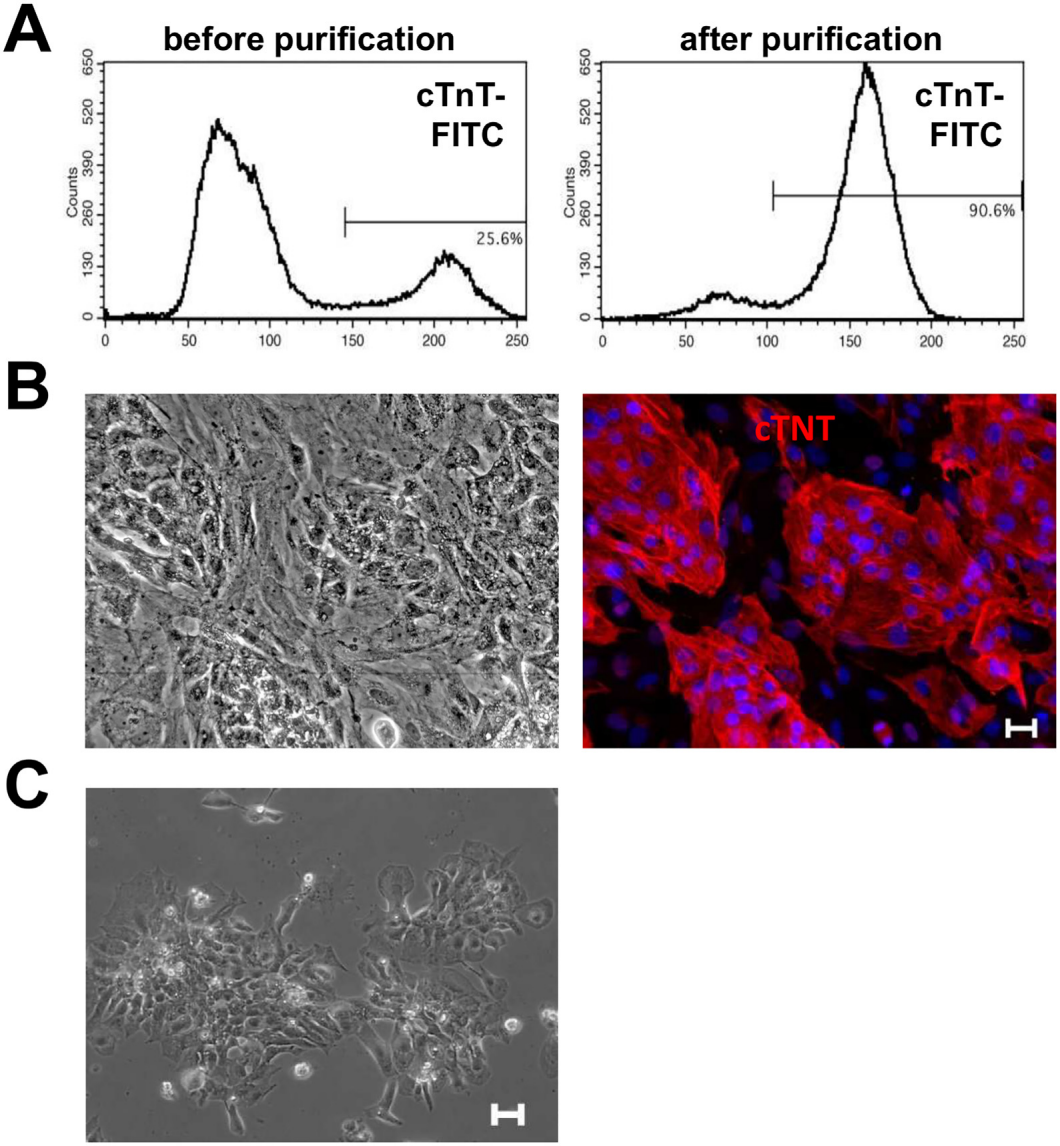
Purification of iPS cell-derived cardiomyocytes with metabolic selection. **A:** Percentage of cTnT-positive cells in suspension culture before and after metabolic selection with lactate medium; **B:** Plated cells from a suspension culture treated with metabolic selection with lactate for 3 days prior plating. Immunofluorescent staining with cardiac troponin T (cTNT) and DAPI highlights the cardiomyocytes (scale bar 25 μm for both); **C:** Purified cardiomyocytes after additional metabolic selection with lactate medium for 8 days after plating (scale bar 50 μm).

Therefore, a recently published purification strategy based on a restrictive medium was tested [22]. This approach takes advantage of the cardiomyocytes’ distinct metabolic capabilities. Several batches of iPS cell-derived cardiomyocytes were exposed to glucose-depleted lactate medium from day 16 on forward. Prior to lactate exposure, the cardiomyocyte purity of a given culture was determined via flow cytometry for cTnT marker expression. After 5–7 days of lactate purification, cardiac enrichment was verified through cTnT marker expression using flow cytometry. With starting purities between 30–45%, > 90% pure cardiomyocytes could be generated after 5–7 days of exposure to lactate (Fig 3A, Table 1, S3 Table). In addition, determined cell counts and cTnT marker expression before and after lactate treatment verified that the cells lost during this process were mainly contaminating cells and that only 50–65% of cardiomyocytes were lost (Table 1, S3 Table).

The purified cardiomyocytes were subsequently plated and maintained without significant purity losses. Cultures containing < 30% cardiomyocytes pre lactate treatment will not reach the aforementioned purities within 7 days of lactate treatment (data not shown). Prolonged exposure to lactate resulted in excessive cell clumping and cell death. Therefore we added a second lactate purification to purify batches of cardiomyocytes with < 80% cTnT positive cells after 7 days of metabolic selection. The cells were plated onto gelatin-coated plates followed by a second lactate treatment as a monolayer for up to 5 days. Fig 3B gives an example for plated cells before the second lactate purification step (see also S3 and S4 Movies). Staining with anti-cTnT antibody demonstrates the cardiomyocytes content in the cell monolayer. After 5 days of lactate exposure, only islands of beating clusters comprised of cardiomyocytes remained on the plates (Fig 3C and S5 Movie) and were available for subsequent phenotypic characterization.

### Functional characterization of iPS cell-derived cardiomyocytes

**Fig 4:**
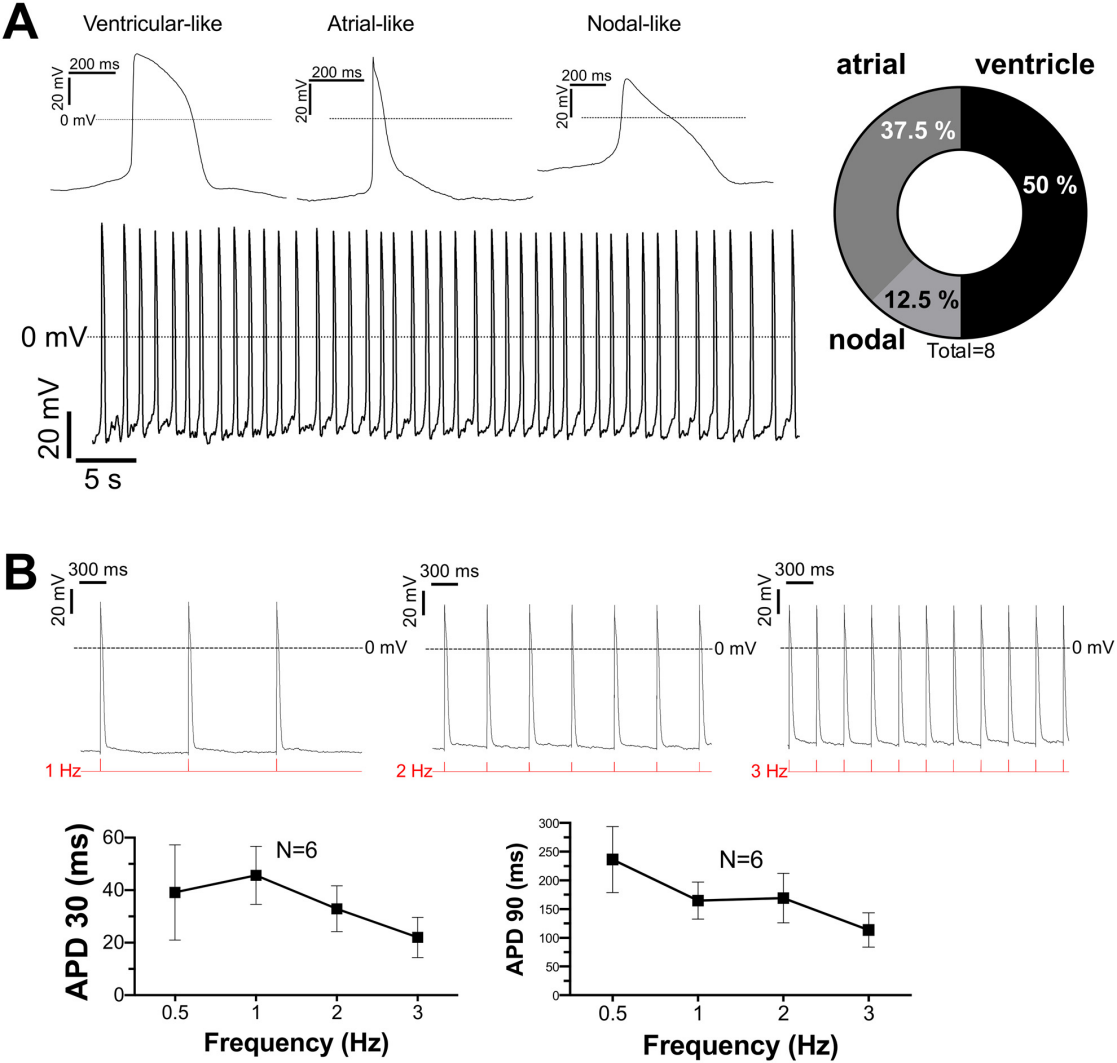
Action potentials characteristics of iPS cell-derived cardiomyocytes. **A:** Left panel shows exemplary original traces of APs of spontaneously contracting cardiomyocytes. Distinct AP morphologies representing ventricular-, atrial-, and nodal-like cardiomyoctes were discriminated. Right panel shows the proportional distribution.** B:** Original traces of APs induced by current injection (current clamp) at various frequencies. Interestingly, iPS cell-derived cardiomyocytes responded to stimulation rates as high as 3 Hz. Mean data for AP duration at 30% and 90% repolarization (APD30 and 90, respectively) showed a rate-dependent shortening in steady-state AP duration.

In order to investigate the electrical activity of differentiated cardiomyocytes, whole-cell patch clamp technique was performed. Fig 4A shows original traces of spontaneous actions potentials (APs) recorded in current clamp mode. In the population of cells investigated (n = 8), 50% of the cells displayed ventricular-like APs, while 37.5% had atrial-like AP and only 12.5% showed action potentials that are consistent with nodal cardiomyocytes. Detailed analysis of resting membrane potential (RMP), action potential amplitude (APA), maximal AP upstroke velocity (APA) and action potential duration at 30%, 50%, 80% and 90% repolarization (APD 30, 50, 80 and 90) are shown in Table 2. For a subgroup of investigated cardiomyocytes, APs were elicited using square current pulses at variable frequency (0.5–3 Hz). As shown in Fig 4B, a frequency dependent shortening of the steady-state AP duration could be observed in the iPS cell-derived cardiomyocytes. The underlying mechanism of rate-dependent shortening of AP duration includes an increase in slow delayed rectifier potassium current, I_Ks_, [34] and increased Na-Ca exchanger current [35]. This suggests that the investigated cardiomyocytes may express a functional IKs and Na-Ca exchanger current.

**Table 2:** AP characteristics of iPS cell-derived cardiomyocytes.

APs (n = 8)	C_m_ (pF)	SAF (Hz)	MDP (mV)	APA (mV)	Max dV/dt (V/s)	APD_30_ (ms)	APD_50_ (ms)	APD_80_ (ms)	APD_90_ (ms)
Ventricular-like (n = 4)	99.2 ± 29.4	0.5 ± 0.1	-45.0 ± 9.6	77.1 ± 12.7	11.3 ± 4.7	336.9 ± 116.4	414.8 ± 144.0	605.3 ± 137.5	737.7 ± 95.9
Nodal-like (n = 3)	257.7 ± 86.0	0.6 ± 0.3	-35.3 ± 1.1	55.6 ± 4.0	1.7 ± 0.6	556.9 ± 170.4	817.0 ± 311.1	1109 ± 433.9	1187.0 ± 466.0
Atrial-like (n = 1)	36.0	1.1	-50.1	87.2	20.7	55.0	69.0	114.0	177.0

Data are mean ± SEM. n indicates the cell number; C_m_: membrane capacitance; SAF: spontaneous AP frequency; MDP: maximum diastolic potential; APA: AP amplitude; max dV/dt: maximum rate of rise of the AP upstroke; APD30/APD50/APD80: AP duration measured at 30%, 50%, 80% or 90% repolarization, respectively.

**Fig 5:**
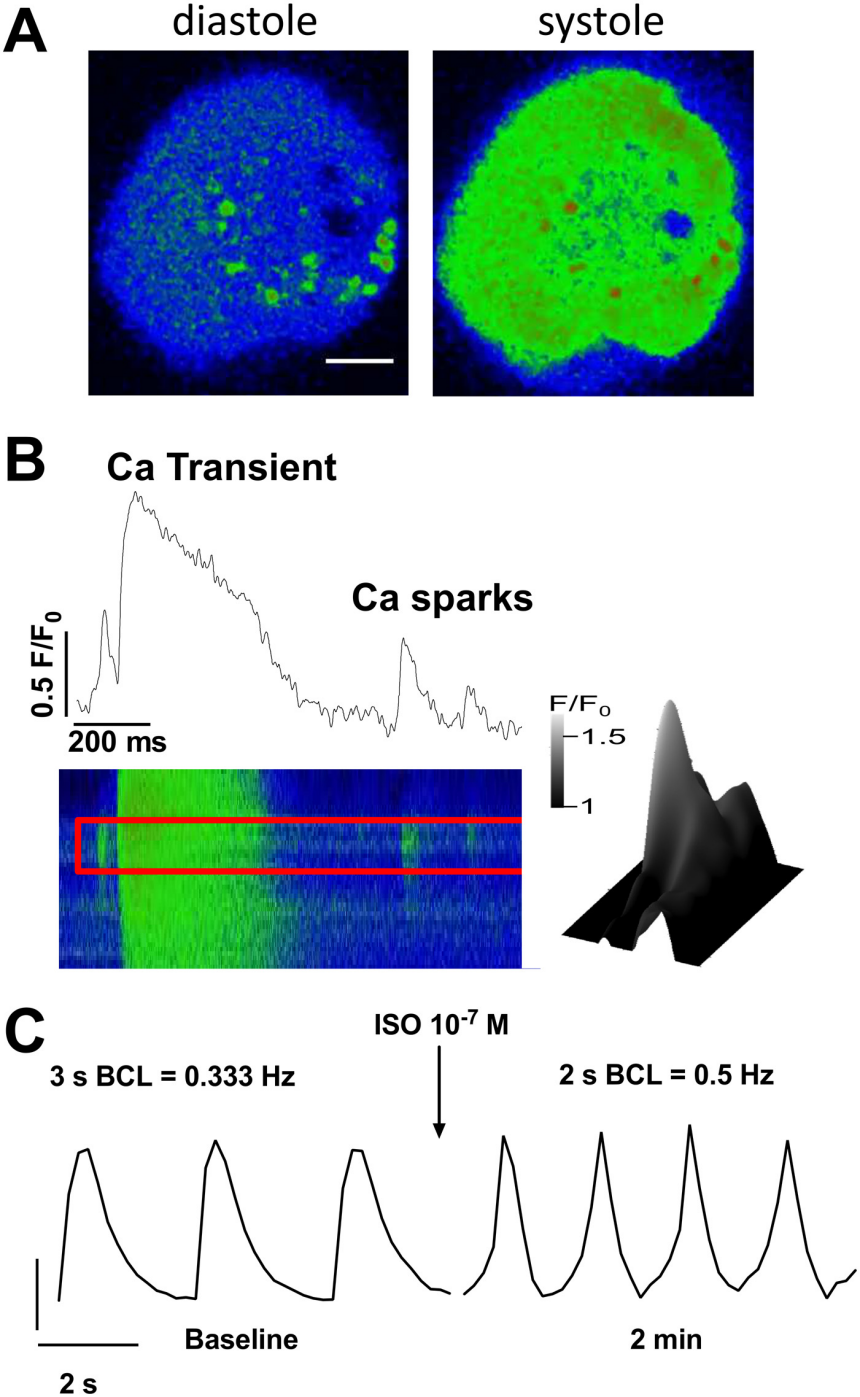
Spontaneous intracellular Ca fluctuations in iPS cell-derived cardiomyocytes. **A:** Exemplary traces of fluo-4 fluorescence in an iPS cell-derived cardiomyocyte at low-diastolic (left) and high-systolic [Ca]_I_ (right). Data was acquired in frame-scan mode (scale bar 10 μm). **B:** Original recording of a fast line-scan image (lower panel) and derived fluo-4 fluorescence intensity as a function of time (upper panel). A typical Ca transient is visible. In addition, spontaneous and localized diastolic Ca release, i.e. Ca sparks, are detectable. Lower right panel shows a surface plot of a Ca spark. **C:** Original recordings of spontaneous Ca transients (acquired in line-scan mode) at baseline and upon exposure to isoproterenol. Spontanous Ca transient frequency increased from 0.33 to 0.5 Hz.

Intracellular Ca was measured in iPS cell-derived cardiomyocytes loaded with the Ca indicator Fluo-4 AM (10 μmol/L, 10 min). Fig 5 shows original traces acquired in frame scan (A) and line scan (B) mode. Original recordings of the dynamic changes in intracellular Ca are also shown using high frequency frame scan mode (S6 Movie). Corresponding to spontaneous action potentials, the iPS cell-derived cardiomyocytes show spontaneous rhythmic fluctuations of the intracellular Ca concentration, i.e. Ca transients, indicative of functional excitation-contraction-coupling [36]. Moreover, the spontaneous frequency and amplitude of these Ca transients were increased in the presence of the β_1_-receptor agonist isoproterenol (10^–7^ mol/L; Fig 5B). Isoproterenol also significantly enhanced the decay time of the Ca transient. This suggests that the iPS cell-derived cardiomyocytes express β_1_-receptors and downstream mediators required for the positive chronotropic, inotropic and lusitropic response.

**Fig 6:**
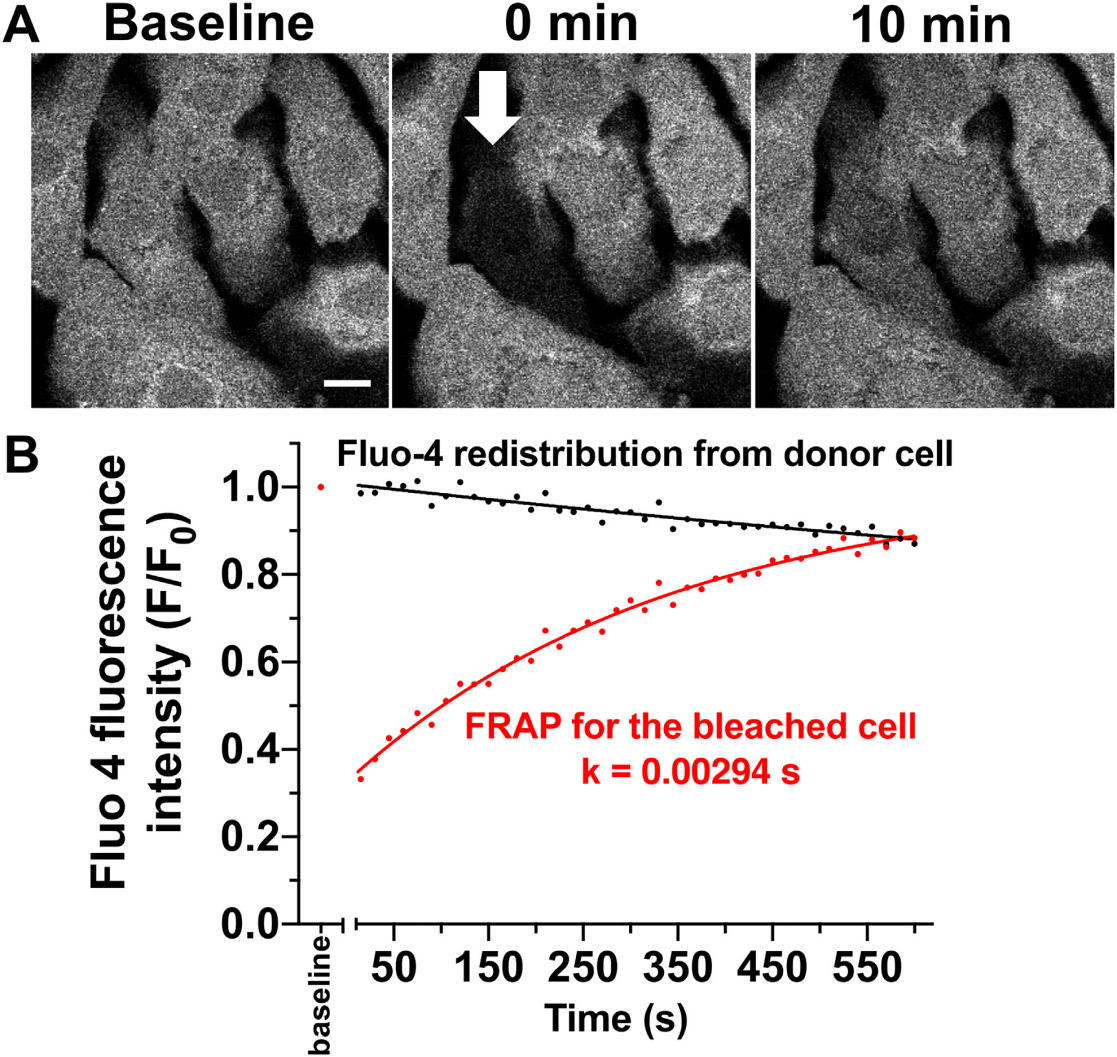
Functional gap junctions in cardiac clusters. **A:** Original recording of a iPS cell-derived cardiomyocyte cluster loaded with the gap junction permeable dye fluo-4 at baseline (left panel, scale bar 10 μm), immediate after photobleaching of a single cell (mid panel, arrow) and 10 minutes later (right panel). **B:** Analysis of fluorescence intensity as a function of time at two regions of interest: the bleached cell and an adjacent donor cell. The fluorescence recovery after photobleaching (FRAP) could be fit with a single exponential with a recovery rate constant k of 2.94 ms. The simultaneous drop in fluorescence in the adjacent donor cell that could also be fit with a single exponential (k = 0.51 ms).

Interestingly, iPS cell-derived cardiomyocytes also showed spatially and temporally randomly distributed spontaneous Ca release events, called Ca sparks (Fig 5B). The latter occurs due to spontaneous diastolic re-openings of cardiac ryanodine receptor clusters [36] and their presence indicates a functional sarcoplasmic reticulum typically found in mature cardiomyocytes. Analysis of fluorescence recovery after photobleaching confirmed the presence of functional gap junctions (Fig 6). Interestingly, the rate constant of fluorescence recovery was very similar to previously published data (k = 0.5/min)[30].

## Discussion

Induced pluripotent stem (iPS) cell-derived cardiomyocytes are an important tool for investigating disease model systems, regenerative medicine or drugs [17]. In initial studies, iPS-derived cardiomyocytes originated from dermal fibroblasts [37–41]. In order to use more easily accessible starting material, efforts were undertaken to obtain iPS from peripheral blood mononuclear cells (PBMC)[4, 42–44]. In this study, the iPS cells serving as the starting material for cardiac differentiation were very reproducibly generated from human PBMC, albeit the reprogramming efficiencies were relatively low ranging at 0.0001 to 0.002%. Microscopic and molecular analyses revealed that all generated iPS cell lines had ES-like morphological characteristics and were of high quality. This was evident from uniform expression of alkaline phosphatase and high levels of pluripotent marker expression (> 95%). These data are in line with reports of successful reprogramming of human peripheral blood cells into iPS cells [42, 43].

To efficiently and robustly differentiate these iPS cells into cardiomyocytes, previously developed protocols were optimized [12, 45]. In brief, to induce mesodermal specification, activin A, BMP-4 and bFGF were added at defined time points and concentrations during the differentiation process mimicking primitive streak formation and germ layer induction *in vivo*. Combining this directed differentiation approach with hypoxic growth conditions resulted in a cardiomyocyte yield of 30–45% around day 16. These cardiomyocytes beat spontaneously in culture and expressed the appropriate structural markers cTnT, α -actinin, N cadherin, connexin 43, and MLC2v as determined through protein and gene expression studies. Interestingly, the expression of connexin 43 correlates with the presence of functional gap junctions, being further confirmed by FRAP analysis (Fig 6).

Although cardiomyocytes were generated from PBMC derived-iPS cells in the above-mentioned studies, purification strategies were either not pursued or focused on antibiotic selection, which required tedious upfront genetic engineering efforts [19].

At the beginning of this investigation, MACS-based purification was attempted as a strategy for cardiomyocyte enrichment and purification. Dubois et al. and Uosaki et al. previously showed that specific cell surface marker can be used to reach cardiomyocyte levels >95% [16, 20]. Although in our experiments MACS-based positive selection with either SIRPA or VCAM1 increased cardiomyocyte purity from about 36% to 80% and 89% respectively, up to 80% of cardiomyocytes were lost during this selection procedure. MACS-based depletion with CD90 and CD140b also resulted in a twofold increase of cardiomyocyte content, but did not enable cardiomyocyte purification beyond 50% with again high losses of cardiomyocytes (Table 1). Substantial cell loss was also reported by Dubois et al. [16] for magnetic bead sorting with SIRPA. Uosaki et al. [20] did not specify the cell loss for magnetic bead sorting with VCAM1. In addition, we observed a drastically reduced viability post sorting. The MACS-purified cardiomyocytes did not attach well and failed to beat indicative of compromised cellular fitness. Overall, considering that MACS-based purification requires expensive reagents, such as large quantities of labeling antibodies, beads and sorting columns, this method of purification was not further pursued as a viable cardiac purification option. To our knowledge, the reports by Dubois et al. and Uosaki et al. remain the only ones demonstrating successful MACS-based purification of cardiomyocytes.

Besides MACS-based enrichment, cardiomyocytes can also be purified using restrictive media with an essentially glucose free lactate supplemented medium [22]. This approach takes advantage of the fact that cardiomyocytes can survive and function properly in the absence of glucose by lactate metabolization via oxidative phosphorylation, while most other cell types cannot. Using the lactate-based purification method, it was possible to enrich cardiomyocytes to > 90% after 5–7 days of lactate medium exposure as based on flow cytometry analysis for cTnT positive cells. Cardiomyocyte losses were substantially lower (<50%) when applying this selection strategy compared to MACS-based purification (>70%). However, to achieve final cardiomyocyte purity of > 90%, the cardiomyocyte content on day 17–20 prior to lactate treatment had to be at least 40%. By plating cell populations with less than 80% pure cardiomyocytes onto gelatin-coated dishes followed by 3–5 days of additional lactate treatment beginning 2 days post-plating, we developed a method for further purifying cultures that would otherwise eventually been discarded due to overgrowing contaminating cells.

Electrophysiological characterization confirmed that the cardiomyocytes derived from iPS cells exhibited similar action potential characteristics found in more mature cardiomyocytes. Functional experiments revealed spontaneous action potentials and Ca transients indicating an intact excitation-contraction coupling. The shape and morphology of the AP corresponds to a cell population consisting of 50% ventricular cells, 37.5% atrial und 12.5% nodal cells. Moreover, we found evidence for the presence of a functional sarcoplasmic reticulum (i.e. Ca sparks) and an intact β_1_-adrenergic response. Finally, we could show a frequency-dependent acceleration of repolarization and a response to stimulation rates as high as 3 Hz. To our knowledge, this is the first demonstration of iPS cell-derived cardiomyocytes capable of rhythmic activity at such a high frequency in vitro.

The robust generation of large quantities of highly pure cardiomyocytes is crucial for their subsequent application in disease modeling, drug discovery and toxicity testing as well as regenerative medicine. Previously, the utility of iPS-cell derived cardiomyocytes in monogenetic disease modeling has been demonstrated [37–39, 46–48]. Many of these diseases are caused by mutations in ion channel encoding genes and can be recapitulated *in vitro* by performing electrophysiological characterization studies on a small quantity of single cardiomyocytes. However, to develop suitable *in vitro* disease models for conditions resulting from mutations in sarcomeric and/or cytoskeletal genes which are involved in force generation and transmission respectively, large numbers of purified cardiomyocytes grown as cardiac cell sheets are needed.

In the field of regenerative medicine, three dimensional sheets comprised of human iPS cell-derived cardiomyocytes have already been developed in efforts to address the need for new therapies for heart disease [49]. However, their delivery to the heart as well as their integration into the diseased tissues still poses significant hurdles. In addition, iPS cell-derived cardiomyocytes also show large potential as a tool for drug discovery and toxicity testing [50]. For the adaptation of iPS-cell derived cardiomyocytes for these applications, large quantities of highly pure cells recapitulating normal human cardiac biology *in vitro* and meeting reproducibility requirements are necessary.

Our combined protocol for a highly robust and scalable cardiac differentiation and purification can provide a simple and cost-efficient method to yield the necessary cardiomyocyte numbers.

## Conclusion

We developed a novel protocol to generate highly purified human cardiomyocytes, which were derived from iPS cells originating from peripheral blood mononuclear cells. It could be demonstrated that the purity of the differentiated cell population improved dramatically to more than 90% cardiomyocytes. Our protocol combines a non-invasive source for generation of iPS cells from PBMC, an optimized directed cardiac differentiation, and a purification based on lactate medium. The latter neither requires laborious genetic manipulations nor expensive laboratory equipment or reagents, but instead provides a simple, cost-efficient method to generate large numbers of highly pure functional cardiomyocytes. This combined protocol will foster the use of iPS cell-derived cardiomyocytes for disease modeling, drug discovery, and regenerative medicine.

## Supporting Information

S1 Movie Beating aggregates on day 39 after start of differentiation without metabolic selection. Scale bar represents 200 μm.
(MOV)

S2 Movie Beating aggregates on day 26 after start of differentiation without metabolic selection. Scale bar represents 200 μm.
(MOV)

S3 Movie Plated cells on day 35 after start of differention before the second round of metabolic selection. Scale bar represents 100 μm.
(MOV)

S4 Movie Plated cells on day 35 after start of differention before the second round of metabolic selection. Scale bar represents 100 μm.
(MOV)

S5 Movie Beating cardiomyocytes after metabolic selection for nine days. Scale bar represents 100 μm.
(MOV)

S6 Movie Ca transients and diastolic Ca sparks. Fluo-4 loaded cardiomyocytes show rhythmic fluctuations in the cytosolic Ca concentration, i.e. Ca transients. Moreover, during diastole, spontaneous elementary Ca release events from the sarcoplasmic reticulum, i.e. Ca sparks, are visible.
(MOV)

S1 Table List of pre-designed TaqMan assays. (DOCX)

S2 Table Cardiomyocyte yields after enrichment by MACS positive selection or depletion. (DOCX)

S3 Table Cardiomyocyte yields after purification with lactate metabolic selection. (DOCX)
