# Supplementary material for: Generation of Highly Purified Human Cardiomyocytes from Peripheral Blood Mononuclear Cell-Derived Induced Pluripotent Stem Cells
Source: PLoS One. 2015 May 13;10(5):e0126596. doi: 10.1371/journal.pone.0126596 (PMC4430251; doi:10.1371/journal.pone.0126596)
Supplement: S1 Table — (DOCX) [file pone.0126596.s007.docx]

**S1 Table.** **List of pre-designed TaqMan assays.**

| **Gene** | **Assay type** | **Assay ID** | **Gene Symbol** | **Gene Name** |
| --- | --- | --- | --- | --- |
| B2M | Reference gene | Hs00984230_m1 | B2M | beta-2-microglobulin |
| TNNT2 | Target | Hs00165960_m1 | TNNT2 | troponin T type 2 (cardiac) |
| MYL2 | Target | Hs00166405_m1 | MYL2 | myosin, light chain 2, regulatory, cardiac, slow |
| MYL2 | Target | Hs01125721_m1 | MYL2 | myosin, light chain 2, regulatory, cardiac, slow |

Pre-designed TaqMan assays were used for quantitative PCR analysis of cardiac specific markers. Assays were purchased from Life Technologies.
