# Supplementary material for: Generation of Highly Purified Human Cardiomyocytes from Peripheral Blood Mononuclear Cell-Derived Induced Pluripotent Stem Cells
Source: PLoS One. 2015 May 13;10(5):e0126596. doi: 10.1371/journal.pone.0126596 (PMC4430251; doi:10.1371/journal.pone.0126596)
Supplement: S2 Table — (DOCX) [file pone.0126596.s008.docx]

**S2 Table. Cardiomyocyte yields after purification with lactate metabolic selection.**

|  | **before enrichment/purification** | | | **after enrichment/purification** | | | **cells lost in %** | |
| --- | --- | --- | --- | --- | --- | --- | --- | --- |
|  | **total cells** | **% cTNT+** | **cTNT+ cells** | **total cells** | **% cTNT+** | **cTNT+ cells** | **total cells** | **cTNT+ cells** |
| Cell line 1 | 3.7 x 10^6^ | 42.3 | 2.8 x 10^6^ | 1.5 x 10^6^ | 80.1 | 1.2 x 10^6^ | 77.0 | 56.4 |
| Cell line 1 | 1.3 x 10^7^ | 45.2 | 5.8 x 10^6^ | 3.4 x 10^6^ | 87.2 | 3.0 x 10^6^ | 73.0 | 48.0 |
| Cell line 2 | 1.3 x 10^7^ | 39.2 | 5.0 x 10^6^ | 1.9 x 10^6^ | 87.1 | 1.7 x 10^6^ | 85.0 | 66.7 |
| Cell line 2 | 4.2 x 10^7^ | 45.9 | 1.9 x 10^6^ | 9.5 x 10^6^ | 91.0 | 8.6 x 10^6^ | 77.4 | 55.1 |
| mean ± SEM | 1.9 x 10^7^ ±6.9 x 10^6^ | 43.1 ± 1.3 | 8.2 x 10^6^ ± 3.2 x 10^6^ | 4.1 x 10^6^ ± 1.6 x 10^6^ | 86.4 ± 2.0 | 3.65 x 10^6^ ± 1.5 x 10^6^ | 78.1 ± 2.2 | 56.6 ± 3.3 |

Total cell counts and cTnT positive cell counts (cTnT+ cells) were calculated per T75 flask.
