# Supplementary material for: Generation of Highly Purified Human Cardiomyocytes from Peripheral Blood Mononuclear Cell-Derived Induced Pluripotent Stem Cells
Source: PLoS One. 2015 May 13;10(5):e0126596. doi: 10.1371/journal.pone.0126596 (PMC4430251; doi:10.1371/journal.pone.0126596)
Supplement: S3 Table — (DOCX) [file pone.0126596.s009.docx]

**S3 Table. Cardiomyocyte yields after enrichment by MACS positive selection or depletion.**

|  |  | **before enrichment/purification** | | | **after enrichment/purification** | | | **cells lost in %** | |
| --- | --- | --- | --- | --- | --- | --- | --- | --- | --- |
|  |  | **total cells** | **% cTNT+** | **cTNT+ cells** | **total cells** | **% cTNT+** | **cTNT+ cells** | **total cells** | **cTNT+ cells** |
| SIRPA | Cell line 1 | 1.7 x 10^7^ | 21.8 | 3.7 x 10^6^ | 9.3 x 10^5^ | 71.9 | 6.7 x 10^5^ | 94.5 | 81.9 |
| SIRPA | Cell line 1 | 1.1 x 10^7^ | 23.4 | 2.6 x 10^6^ | 1.8 x 10^7^ | 61.1 | 1.1 x 10^6^ | 83.6 | 57.3 |
| SIRPA | Cell line 2 | 1.1 x 10^7^ | 27.3 | 3.0 x 10^6^ | 7.0 x 10^6^ | 72.3 | 5.1 x 10^5^ | 93.6 | 83.2 |
| SIRPA | Cell line 2 | 9.4 x 10^6^ | 36.0 | 3.4 x 10^6^ | 9.0 x 10^5^ | 80.2 | 7.2 x 10^5^ | 90.4 | 78.6 |
| SIRPA  mean ± SEM |  | 1.2 x 10^7^ ±1.5 x 10^6^ | 27.1 ± 2.8 | 3.2 x 10^6^ ± 2.1 x 10^5^ | 1.1 x 10^6^ ± 2.1 x 10^5^ | 71.4 ± 3.4 | 7.5 x 10^5^ ± 1.1 x 10^5^ | 90.6 ± 2.1 | 75.3 ± 5.2 |
| VCAM1 | Cell line 1 | 5.9 x 10^6^ | 34.1 | 2.0 x 10^6^ | 3.6 x 10^5^ | 55.6 | 2.0 x 10^5^ | 93.8 | 89.9 |
| VCAM1 | Cell line 1 | 1.7 x 10^7^ | 21.8 | 3.7 x 10^6^ | 4.0 x 10^5^ | 68.4 | 2.7 x 10^5^ | 97.6 | 92.6 |
| VCAM1 | Cell line 2 | 6.8 x 10^6^ | 36.3 | 2.5 x 10^6^ | 5.0 x 10^5^ | 89.1 | 4.5 x 10^5^ | 92.6 | 82.0 |
| VCAM1 mean ± SEM |  | 9.9 x 10^6^ ± 2.9 x 10^6^ | 30.7 ± 3.7 | 2.7 x 10^6^ ± 4.1 x 10^5^ | 4.2 x 10^5^ ± 3.3 x 10^4^ | 71.0 ± 8.0 | 3.1 x 10^5^ ± 5.9 x 10^4^ | 94.7 ± 1.2 | 88.2 ± 2.6 |
| CD90/140b | Cell line 1 | 9.3 x 10^6^ | 23.3 | 2.2 x 10^6^ | 1.4 x 10^6^ | 49.8 | 6.8 x 10^5^ | 85.2 | 68.3 |
| CD90/140b | Cell line 1 | 1.1 x 10^7^ | 23.4 | 2.6 x 10^6^ | 3.0 x 10^6^ | 37.9 | 1.1 x 10^5^ | 72.7 | 55.8 |
| CD90/140b mean ± SEM |  | 1.0 x 10^7^ ±6.1 x 10^5^ | 23.4 ± 0.0 | 2.4 x 10^6^ ± 1.5 x 10^5^ | 2.2 x 10^6^ ± 5.7 x 10^5^ | 43.9 ± 4.2 | 9.1 x 10^5^ ± 1.6 x 10^5^ | 78.9.± 4.4 | 62.1 ± 4.4 |

SIRPA or VCAM1 were used for MACS positive selection whereas CD90 and CD140b were used for MACS depletion. Total cell counts and cTnT positive cell counts (cTnT+ cells) were calculated per T75 flask.
